# DPABI harmonization: A toolbox for harmonizing multi-site brain imaging for big-data era

**DOI:** 10.1162/imag_a_00388

**Published:** 2024-12-13

**Authors:** Yu-Wei Wang, Han-Lin Wang, Chao-Gan Yan

**Affiliations:** CAS Key Laboratory of Behavioral Science, Institute of Psychology, Beijing, China; Department of Psychology, University of Chinese Academy of Sciences, Beijing, China; International Big-Data Center for Depression Research, Institute of Psychology, Chinese Academy of Sciences, Beijing, China; Department of Psychiatry, Yale University School of Medicine, New Haven, CT, United States; Sino-Danish Center, University of Chinese Academy of Sciences, Beijing, China; Magnetic Resonance Imaging Research Center, Institute of Psychology, Chinese Academy of Sciences, Beijing, China; Department of Psychological and Cognitive Sciences, Tsinghua University, Beijing, China

**Keywords:** data processing, quality control, resting-state fMRI, standardization, statistical analysis

## Abstract

Pooling multi-site datasets is the dominant trend to expand sample sizes in neuroimaging field, thereby enhancing statistical power and reproducibility of research findings. Nevertheless, the heterogeneity derived from aggregating data from various imaging sites obstructs efficient inferences. Our recent study thoroughly assessed methods for harmonizing multi-site resting-state fMRI images, accelerating progress and providing initial application instructions. Despite this advancement, the removal of such site effects generally necessitates a certain level of programming expertise. In our effort to streamline the harmonization of site effects using advanced methodologies, we are pleased to introduce the DPABI Harmonization module. This versatile tool, allowing agnostic to specific analysis methods, integrates a range of techniques, including the state-of-the-art Subsampling Maximum-mean-distance Algorithms (SMA, recommended), ComBat/CovBat, linear models, and invariant conditional variational auto-encoder (ICVAE). It equips neuroscientists with an easy-to-use and transparent harmonization workflow, ensuring the feasibility of post-hoc analysis for multi-site studies.

## 1 Introduction

Neuroimaging research calls for bigger datasets in the pursuit of validity and reproducibility (Marek et al., 2022). In consideration of the economic benefit and efficiency, aggregating multi-site data emerges as the optimal approach to achieve sufficiently large datasets. With the growing availability of open data, one might expect a proliferation in multicenter studies. However, this is not the case, primarily due to the variability among datasets originating from different sites, with site-specific variances being the major barrier. The site-wise heterogeneity is attributed not only to data acquisition settings, such as scanner manufacturer, magnetic field strength, coil number, and software versions etc., but also to variations stemming from experimental conditions, environmental factors, and individual differences (Yan et al., 2013). Hence, addressing inter-site variability poses a critical challenge.

Some methods were transferred from bioinformatics, that is, ComBat (Johnson et al., 2007). Yet, before being applied to harmonize resting-state functional Magnetic Resonance Imaging (rs-fMRI) data, these methods have to undergo multifaceted evaluations to ensure their effectiveness. Specifically, it is essential to assess whether they can effectively remove site-specific effects while preserving the essential characteristics of the brain’s phenotypes of interests.

Besides, different techniques are rooted in distinct principles. A basic understanding is indispensable to carry out harmonization practices adequately, taking into account concrete data features, research objectives, and configuration considerations. Some methods are hard to implement toward novices, and in most cases, application needs certain expert knowledge for guidance.

**Fig. 1.**
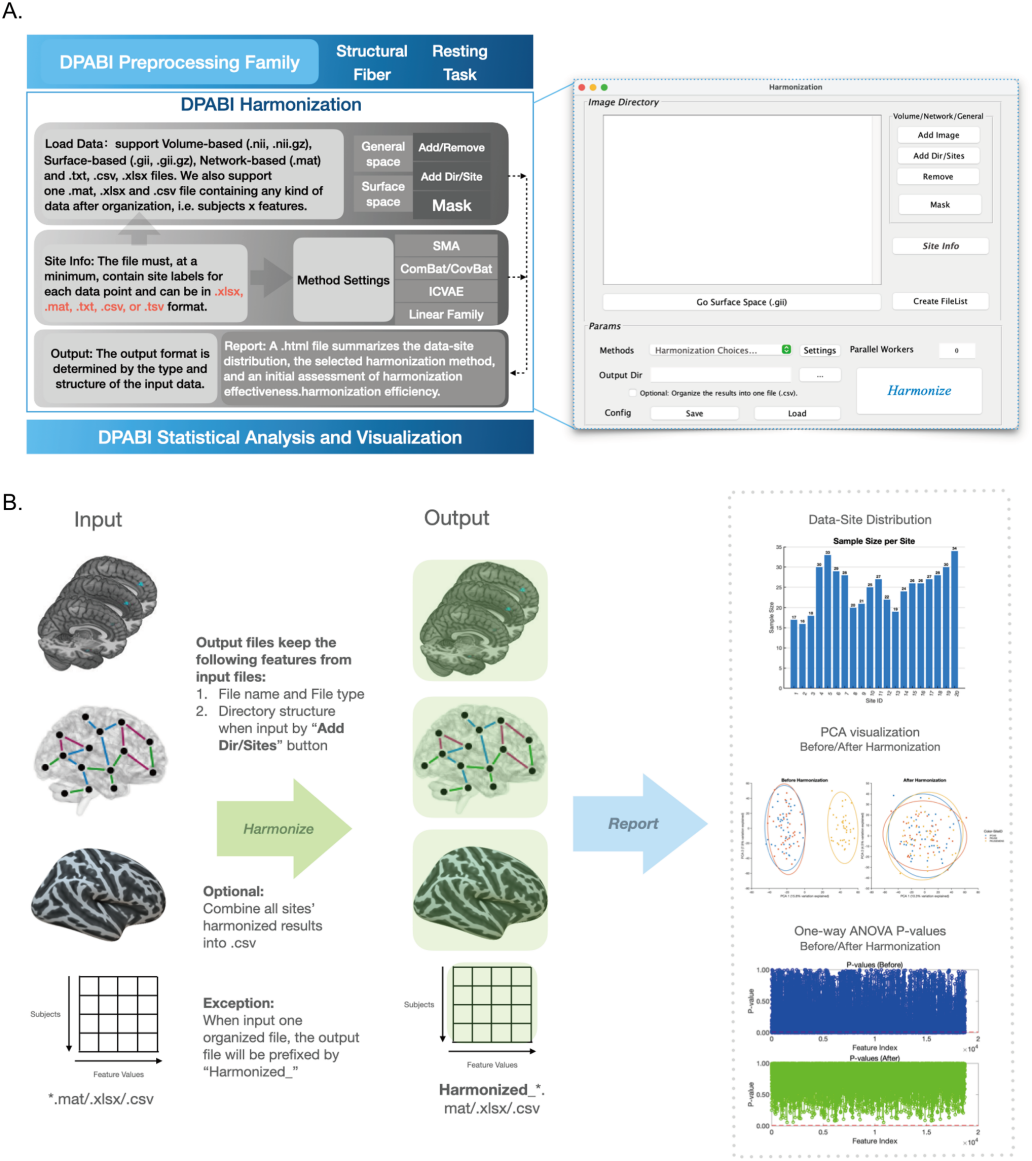
Overview of DPABI harmonization. (A) DPABI Harmonization features, workflow, and main panel. As one independent module of DPABI, DPABI Harmonization can easily bridge the preprocessing pipelines and statistical analysis of DPABI for different modalities. The workflow is straightforward, involving two main steps: 1) Load your data, and 2) Set the approach. Then, click “Harmonize” button and wait for the results and report. (B). The input and output options of DPABI Harmonization, and their corresponding relationship. Input files are consistent with output files in terms of filename and type, and the directory structure is preserved when using “Add Dir/Sites” button to import your data. An exception occurs when you input an organized dataset, in which case the output will also be organized but prefixed “Harmonized_”. If you import multiple files but also want one organized file with all subjects’ harmonized results, just click the “Optional: Organize the results into one file (.csv).” It will additionally output a file named “HarmonizationResults.csv”under your “Output Dir” path, where rows represent subjects and columns represent features.

Recently, we conducted a comprehensive comparison and evaluation of mainstream harmonization techniques on multi-site resting-state magnetic resonance imaging data, providing practical guidance on implementation (Wang et al., 2023). Capitalizing on test-retest datasets, it concludes that SMA is best in effectiveness. These assessments provide primary evidence to navigate harmonization practice. Consequently, to validate the evaluation results and promote the practical application of these methods, we developed an open-source, user-friendly toolkit—Data Processing & Analysis of Brain Imaging (DPABI) Harmonization (Fig. 1). This toolkit integrates these methods, making it more convenient for users to scrutinize past findings, compare various techniques, engage in reproducible research, and select the most suitable approach. To this end, we have expanded the DPABI Harmonization module, incorporating not only the SMA algorithm recommended in our previous study, but also other methods, including ComBat (Fortin et al., 2017,^2018^;Johnson et al., 2007) and CovBat (Chen et al., 2022) (parametric/nonparametric; adjusting/not covariates), invariant conditional variational auto-encoder (ICVAE) (Moyer et al., 2020), as well as the general linear model and linear mixed model.

Thanks to this software’s agnostic framework, researchers can direct their attention to the specific problems at hand. Through our comprehensive analyses, they can discern an optimal route toward harmonization without being time-consuming for grasping a variety of methods and make choices among them, as well as writing programming scripts. They merely need to comprehend the pivotal parameters essential for optimizing harmonization outcomes, all of which we have meticulously elucidated via our user interfaces design.

DPABI Harmonization was developed based on MATLAB2020a (The MathWorks Inc., Natick, MA, US) (RRID: SCR_001622), Statistical Parametric Mapping (Ashburner, 2012) (RRID: SCR_007037), and DPABI (Yan et al., 2016) (RRID: SCR_010501), and adopted Docker (https://docker.com) for python-based ICVAE. It is recommended to use MATLAB version 2020a or later. The required toolboxes inside MATLAB for DPABI Harmonization includes Parallel Computing Toolbox, Statistics and Machine Learning Toolbox, Optimization Toolbox, and Control System Toolbox.

In consistency with DPABI, we fully committed to keeping it open source and permitting third-party modification, tracking the version history using git. Coordinating with other functionalities of DPABI, it mainly oversees the harmonization duty.

In the following section, we will first introduce the parts of the tool and then give detailed guidance on the usage of different methods.

## 2 Functionality

To use this tool for harmonizing multisite datasets, the overall workflow can simply be two steps: load the dataset, ensuring that site information is included; then choose a method that best aligns with your goals. Additional features are available to improve the efficiency, accuracy, and reproducibility of the harmonization process, ensuring a better overall experience.

### 2.1 Data input: spaces and actions

#### 2.1.1 Spaces

To facilitate the harmonization of multi-type neuroimaging data, we define two independent spaces for loading data. As shown inFig. 2, the first is a general data space for voxel-wise brain imaging files (extension as .img, .nii, and .nii.gz), brain networks (.mat or other formats), and any other formats (.xlsx, .csv, .txt). This space is accessible through the main GUI. And the second space is designed for hemispherical-computed brain imaging. It can be reached through button ‘Go Surface Space (.gii)’.

**Fig. 2.**
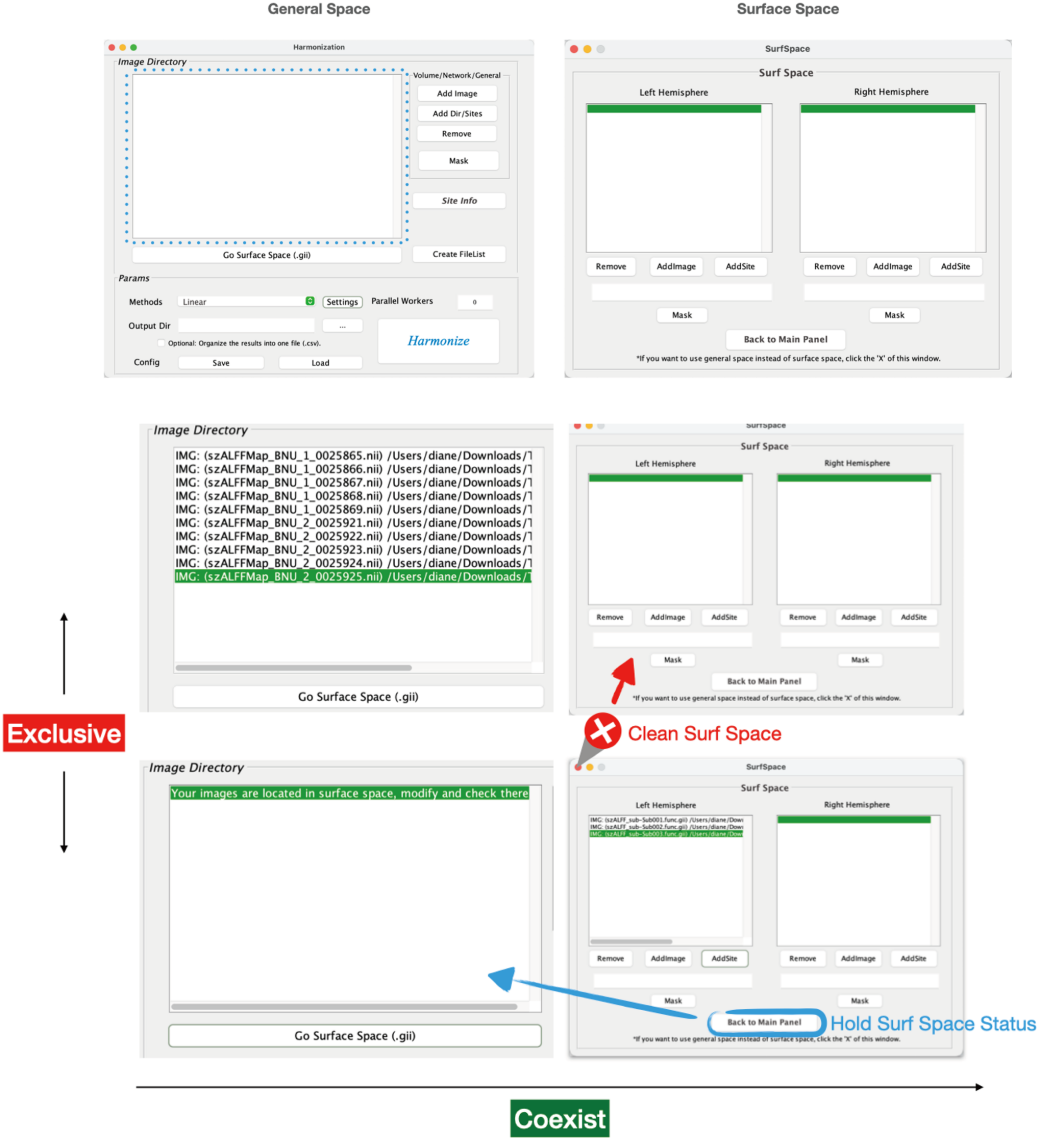
Spaces and their relationship. The first row displays the general space and surface space interfaces. The second and third row shows the different status combinations of them. Rows can coexist but columns are mutually exclusive. When data are located in the general space, the surface space remains empty (second row). Conversely, when you switch to the surface space and load any data, the general space will display the message: “Your images are located in suface space; modify and check there.” (third row).

These two spaces are mutually exclusive (Fig. 2). When it comes to operation, it implies:

1. If the surface space is not empty, you can clear it by clicking the “X” at the top of the window before operating in the general space.
2. When the “Go Surface Space (.gii)” button is pressed and data are loaded into that space, the general space will be cleared, displaying the message: “Your images are located in surface space; modify and check there.”

The two separate spaces are specific for different data. Based on the extension type of the input, both spaces support data files extended by .nii, .nii.gz, .gii, .mat, .xlsx, .csv, .txt. However, for surface space, it only accepts masks in .gii format and cannot read organized data as it does for general space.

#### 2.1.2 Actions

**Fig. 3.**
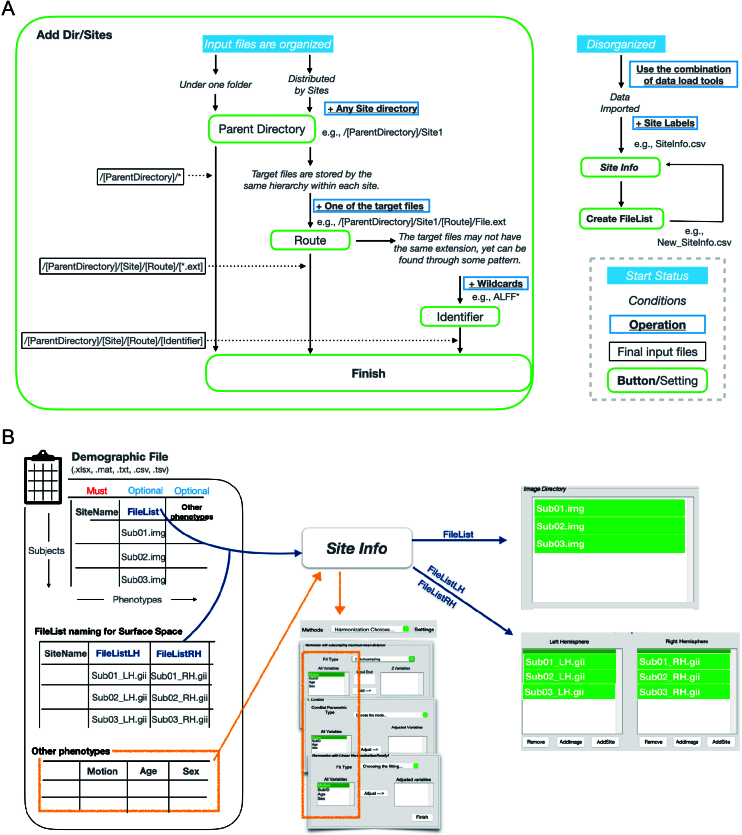
The flow chart of data load. (A) The workflow to import organized and disorganized data. (B) The utility of the “Site Info” button. “*Site Info*” button serves three functions, 1) Retrieve the site labels of each data point (implicit), 2) Supplement “Methods Settings” with phenotypes, and 3) Load data files in alignment with the sequence of sites and other phenotypes.

The method for loading data into each specific space is consistent. In the main panel, you can add data using either the**“Add Image”**button or the**“Add Sites”**button. The**“Add Image”**button allows you to load multiple files from the same directory at once. If brain images are organized within a single directory or distributed consistently by site, you can use the**“Add Sites”**button to import them.Fig. 3Apresents a detailed flowchart illustrating how to use these buttons to add organized data.

1. If brain images are organized within one directory, simply input the directory path into**“Parent Directory”**.
2. If data are organized by sites, ensure that the files for different sites can be accessed via similar paths. For example, if there are two sites, site1 and site2, and the full path for an image at site1 is “..\site1\FunctionalConnectivity\FC_xxx.nii,” then the images for site2 should follow a similar pattern, such as “..\site2\FunctionalConnectivity\FC_xxx.nii.” The folder paths between sites (i.e., site1/site2) and the target files (i.e., FC_xxx.nii) should be consistent, including the names and hierarchy of the directories. To automatically locate your input files, you have two options:
  - If all input files share the same extension and no other files are present in the directory, you can specify one of your target files in the**Route**. The tool will then automatically find and load all files with the same extension.
  - If this is not the case, use the**“Identifier”**with wildcards. For example, to load all files at the end of the route, use “*”. If you want to load files with a specific prefix, include it in the**“Identifier”**. The tool will match the appropriate files based on the wildcard pattern you provide.

If those files are not well-organized, you can use the**“Site Info”**and**“Create FileList”**buttons, or other means, to arrange them systematically in your demographic file. To realize it with our software, first, add the data to the**“Image Directory”**listbox. Then, add your demographic file using**“Site Info”**. Once you have completed these steps, click**“Create FileList”**to generate a new demographic file with an additional**“FileList”**column, which lists the full paths of each file. You can use this column to verify the correspondence between phenotypes and files, and easily load the data next time by adding the updated demographic file to**“Site Info”**.

To delete one chosen image, we can use “Remove” button, and to delete all loaded images, right-click in the list box and clear them.

The “Mask” option is designed to specify which data should be included in the harmonization process. Masks should be binary, containing only 1s and 0s. For voxel-wise and vertex-wise data, the mask must match the size of the input data. Without a mask, all voxels/vertices will be included in the harmonization, which is not recommended. This approach can lead to a large number of zero-value units being processed, which do not contribute to the harmonization and can be inefficient.

For square networks, there is a default mask with the extension .mat. When you input a square matrix, the tool will automatically extract only the upper triangular part of each matrix, excluding the diagonal data. Following harmonization, the output network will consist solely of the harmonized upper triangular portion.

These operations also apply to surface space. You can access the surface space by clicking the**“Go Surface Space (.gii)”**button.

And before going to the method setting section, make sure you have loaded your demographic information through**Site Info**button (Fig. 3B). This must be done ahead of methodology configuration. Besides providing source site classification for each data point, other phenotypes like sex, age etc., will be automatically loaded when you choose a covariate-adjust approach. Also, using a sheet including column named “FileList” for general space or “FileListLH” and “FileListRH” for surface space is another better way to load amounts of files so users can check the correspondence in the sheet to ensure the accuracy of harmonization.

Before proceeding to the method settings, ensure that you have loaded your demographic sheet using the “Site Info” button. This step must be completed before configuring the methodology. Along with specifying source site classifications for each data point, other phenotypes you named across the head of this sheet, such as sex and age, will be automatically displayed in the “All Variables” listbox if you select a covariate-adjusted approach. Furthermore, for a more efficient data-loading process, use a sheet with columns named “FileList” for general space, or “FileListLH” and “FileListRH” for surface space. This approach helps users verify file correspondence with phenotypes and validate the harmonization.

### 2.2 Apply harmonization methods

The toolkit integrates leading methods and organizes them within four sub-interfaces, including SMA, ComBat/CovBat, ICVAE, and Linear. This software manuscript is a follow-up to our previous methodological manuscript (Wang et al., 2023). As the methodological details and choice of harmonization have been deeply discussed in our previous manuscript, we referred the readers to that manuscript and briefly summarized it here.

#### 2.2.1 SMA

##### 2.2.1.1 Logic.

SMA is an approach aiming at solving statistical issues raised by data pooling, through shifting marginal distribution related to the variation of pooling sources to comply with a target source. The naming of SMA encapsulates its two core technical tenets: “subsampling” and “Maximum mean distance” (MMD). The former takes effect in the situations where the data sources (referred to as “sites” here) and other pivotal factors (such as biological characteristics) covary, thereby affecting data distributions. Subsampling is employed to align distributions between the source and target site. In order to get there, the algorithm stratifies samples by key factors in addition to the site, and then iteratively estimates alignment parameters through sampling from subgroups. MMD is exactly the measure quantifying this distance between source and target distributions, in turn influencing parameter estimation to minimize these distances. MMD is a non-parametric method, employing kernel methods, hence allowing assessing distribution similarity in high-dimensional spaces. This feature allows it to deal with complex datasets (e.g., the curse of dimensionality) and robust to changes in scale. In our assessments, SMA excels at both site effect removal and preservation of sufficient biological effects, ensuring identifiability and reproducibility even in challenging scenarios, outperforming alternative methods. Ideal target site for implementing SMA exhibits substantial samples, encompassing a wide range of samples relevant to the research focus. We offer a heuristic formula for selecting the optimal target site from your harmonization datasets. More details are illustrated inWang et al. (2023).

##### 2.2.1.2 Parameter

1. Fit Type: This parameter decides whether considering the influence of phenotypes on data distribution. Choosing “No subsampling” means users assume that only site induces the shift of data distributions, while choosing “Subsampling” implies the complement cases where site and other known factors co-contribute to the differences of distributions.
2. Subsample factors: If and only if “Subsampling”, the covariates (column names in the demographic file except for “SiteName” and “FileList”) shall be displayed in the “All Variables” listbox. Choose those assumed affecting data distribution and set “Zcut” which guides the division of subgroups within each site. To set “Zcut”, input 0 for categorical variables, and input numbers for continuous variables (if there are multiple cuts, using ‘,’ to separate). After that, click “Add—->” to load those variables and cuts to “Z Variables” listbox. If all done, then use “Add to Model: Subgroup” model to finish subgrouping.
3. Target Site: Choosing the target site is a necessary step, no matter subsampling or not.

##### 2.2.1.3 To apply SMA.

**Fig. 4.**
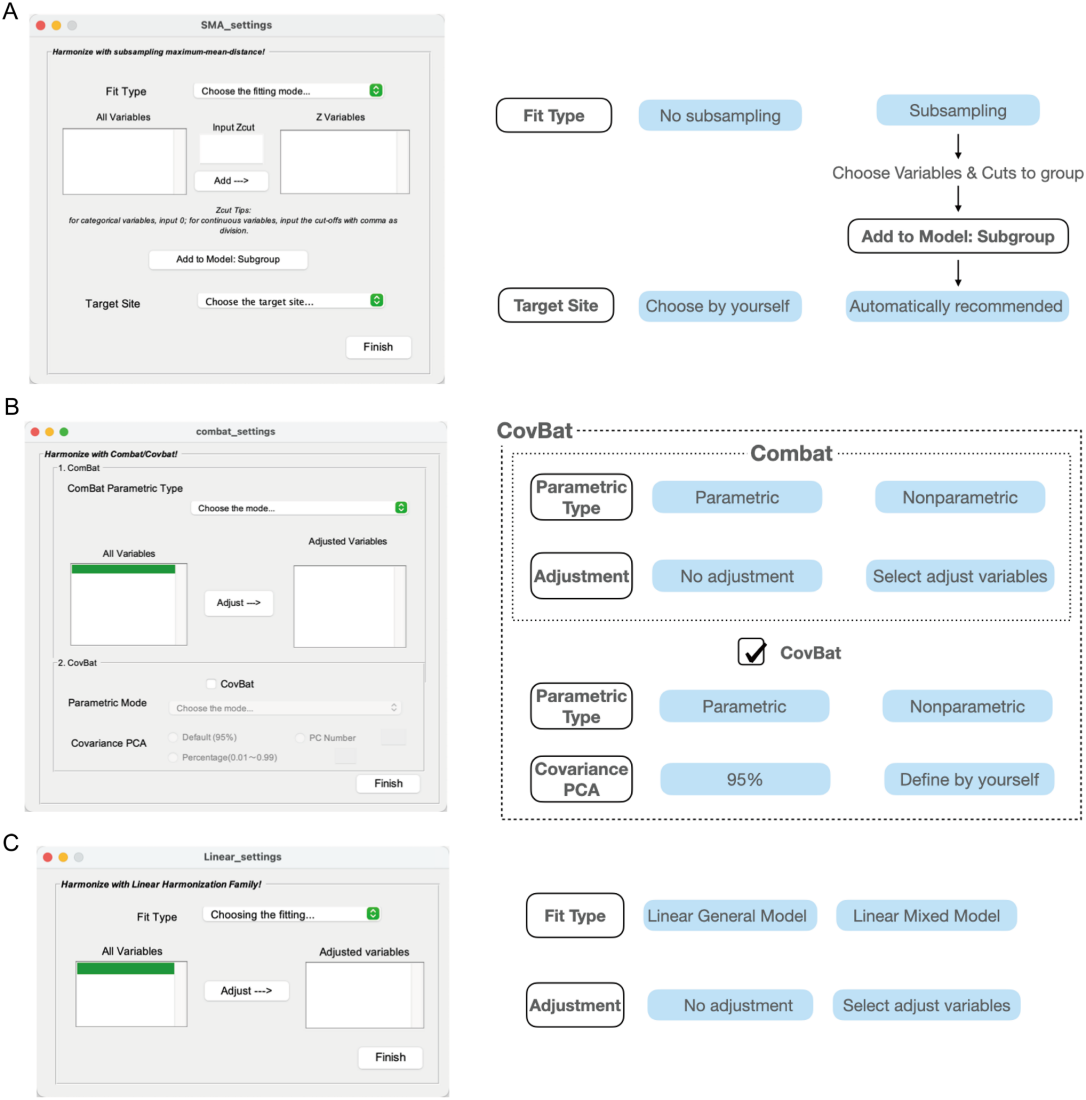
Methodology GUI Settings. The left side of panels (A, B, and C) displays the GUIs for SMA, ComBat/CovBat, and Linear family methods, respectively. The right side of each panel shows the main parameters and their available choices for users. Arrows indicate dependent parameter choices, while the absence of arrows signifies independence between parameters. The parameters that need to be set for ComBat and CovBat are boxed with dotted lines.

Open SMA_settings window (Fig. 4A), the first step is to provide demographic information where site labels for each subject are headed by “SiteName.” Then, choose whether to subsample. If we choose “No Subsampling”, you can directly choose a target site. If we choose “Subsampling”, the variables (headings of the demographic file offered in the first step) will be listed in the listbox “All Variables.” Then, choose the variable and input the cutoff, that is, numbers for continuous variables and 0 for categorical variables. Use the “Add —->” button to load them to “Z variables”. After cutting all needed variables, click the “Add to Model: Subgroup” button, and it will feedback the recommended target site.

#### 2.2.2 ComBat/CovBat

##### 2.2.2.1 Logic.

ComBat (combating batch effects when combining Batches) method was originally introduced from the technology of removing batch effects in genetics (Johnson et al., 2007). It is well known for it adopts the empirical Bayesian to estimate the posterior distribution of site effect so that it achieves removing site effect in mean and variance of site-related variations. CovBat (Chen et al., 2022) is a modified version of ComBat to accommodate machine learning (ML) scenarios by further applying the ComBat principle to removing site effects in covariance.

##### 2.2.2.2 Parameter

###### 2.2.2.2.1 ComBat.

DPABI Harmonization integrates both parametric and nonparametric methods, along with additional adjustment options, to enhance the flexibility of ComBat. The key distinction between parametric and nonparametric lies in the assumptions about the distribution of site effects. If the site effects follow a normal distribution, the parametric approach is recommended due to its speed and computational efficiency. However, if the distribution deviates from normality, the nonparametric option is preferable. Additionally, the adjustment feature allows for the inclusion of other phenotypic variables beyond the site, extracting their linear effects. This ensures that the effects of interest are preserved during harmonization, allowing them to be more prominent in subsequent post-hoc analyses.

###### 2.2.2.2.2 CovBat.

There are four configurable parameters for using CovBat, including the two from ComBat, as the harmonization of mean and variance is identical in both methods. CovBat operates on principles similar to ComBat for selecting between parametric and non-parametric methods; however, while ComBat’s assumptions are centered around mean and variance, CovBat focuses on covariance.

The default setting retains principal components (PCs) that explain 95% of the total variance in the original CovBat R code (https://github.com/andy1764/CovBat_Harmonization/blob/master/R/R/covbat.R). This default is maintained in our implementation. Nevertheless,Chen et al. (2022)conducted a simulation study to better inform the selection of PCs, finding that for a sample size of 100, the optimal site detection accuracy is achieved when CovBat retains PCs, explaining around 90% of the total variance. For a sample size of 250, this threshold increases to 95%. To enhance flexibility, we also offer two additional methods for setting the explained variance: users can specify either the exact number of PCs or the desired percentage of variance explained (0-1).

##### 2.2.2.3 To apply ComBat/CovBat.

After providing demographic information, the first step is to choose the parameters of ComBat (Fig. 4B). ComBat has two parameters: i) parametric/nonparametric settings and ii) whether to perform adjusted regression for interested variables. Users can choose whether to do CovBat by clicking the checkbox after CovBat. CovBat also has two parameters: i) parametric/nonparametric settings and ii) defining how many PCs of covariance should be retained and harmonized. In our paper, we recommended nonparametric ComBat/CovBat with covariates, for CovBat, choose parametric defaultly.

#### 2.2.3 ICVAE

##### 2.2.3.1. Logic.

The concept behind ICVAE involves initially encoding site information and other details separately. The encoder is employed to grasp the distribution characteristics of the original image, abstracting them into a low-dimensional Z vector within an intermediate hidden space. Subsequently, in this low-dimensional space, the site-orthogonal information can be effectively characterized through highly compressed orthogonal dimensions. Upon decoding, these dimensions can be mapped back to the raw data in the same dimensional space. Through controlling intermediate site one-hot encodings, the site information in the generated images can be unified.

##### 2.2.3.2 Parameters.

The structure of ICVAE we implement into DPABI Harmonization is visualized inFig. 5. The input dimension is 512; if the user’s data have a larger dimension of features, the features would be divided accordingly by 512. There are three hidden layers for encoder and decoder, and the loss comprises four parts.$L_{recon}$calculates the distance between output and input to make sure the output has a similar pattern with the input.$L_{Marginal}$is the distance of distributions between encoder and decoder.$L_{prior}$measures the distance between probability density functions of latent space distribution and a standard normal Bayesian prior N(0,I).$L_{adv}$is the accuracy of the prediction of site source for each reconstructed data. The total loss function is as follows,

**Fig. 5.**
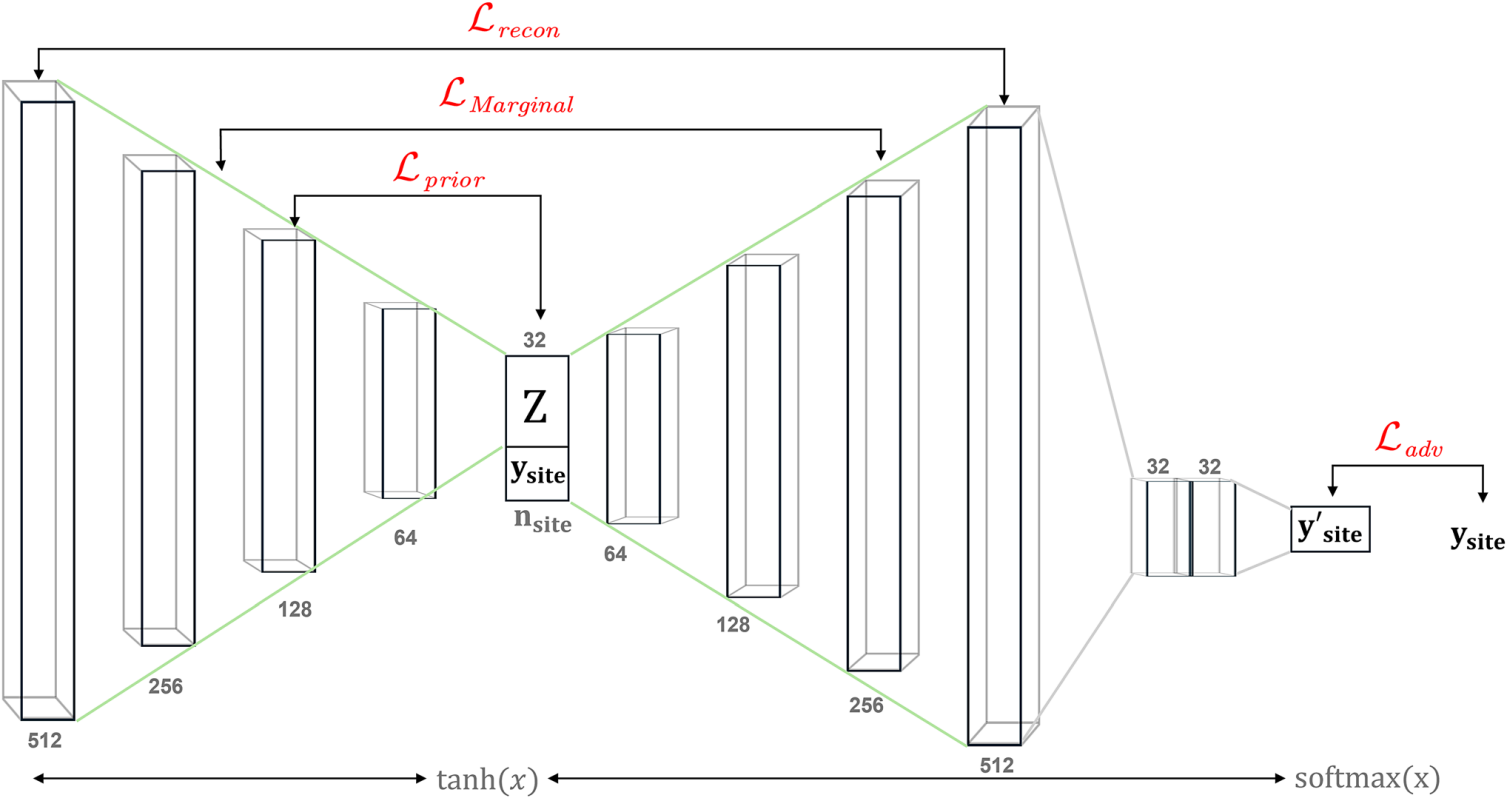
Schematic of the ICVAE Architecture. The ICVAE model is composed of three main components: the encoder, the decoder, and an adversarial neural network. The encoder (to the left of the latent space “Z”) begins with a 512-unit input layer, followed by hidden layers with 256, 128, and 64 units, respectively. The latent space is conditioned on site-specific information, represented by one-hot embeddings with dimensionality. The decoder, positioned to the right of the latent space, mirrors the structure of the encoder. Following the decoder, the adversarial neural network consists of two hidden layers with 32 units each, and an output layer with$n_{site}$units, predicting the probability distribution of the reconstructed output’s source. The training process is driven by a combination of four loss functions: reconstruction loss$L_{recon}$, marginal loss$L_{Marginal}$, prior distribution loss*$L_{prior}$*, and adversarial loss$L_{adv}$. All layers use tanh(x) as the activation function, except the output layer of the adversarial neural network, which employs softmax.

$$L=L_{recon}+\alpha L_{Marginal}+\beta L_{prior}-\gamma L_{adv}$$

whereα,β, andγare hyperparameters to control the weights of loss. Here in our model,α= 1,β= .1,*γ*= 10. Except the activation function of the output of adversarial network being softmax, others are all tanh.

Studies have indicated that VAE-based models, when phenotypes are not considered, may result in the loss of biological information (An et al., 2022;Wang et al., 2023). And VAE-based models retain certain black box characteristics of deep learning that could restrict its application in environments where transparency is crucial, such as clinical trials. Moreover, it is tailored for the harmonization of cross-sectional studies that involve a single batch variable, and it is not suitable for longitudinal studies or datasets where two or more variables contribute to technical noise (Hu et al., 2024). Given the expertise required to train and fine-tune a model for optimal performance on a specific dataset, this method may not yield satisfactory results for many users. Given this, we demonstrate how it can be modified in the following section.

##### 2.2.3.3 To apply ICVAE.

ICVAE is implemented in Python, and to execute it within MATLAB, we use Docker. Before using this method, users must pull the Docker image from Docker Hub to their local system. Once the image is downloaded, users simply need to load the demographic information file and select a target site.

We demonstrate the usage of ICVAE, which has been containerized using Docker. The relevant code can be found under DPABI/RedistributedTools/ICVAE. The construction of the variational auto-encoder and adversarial model are in vae_model.py and adv_model.py, respectively, both of which are called in train.py. Users can modify these files based on their specific needs, such as adjusting the number of layers, the number of cells per layer, training epochs, and hyperparameters.

To continue using the interface we developed after modifying the code, follow these steps to build and push your customized Docker image:

cd your/path/to/modified/ICVAE
docker login
docker build -t your_docker_username/icvae.
docker push your_docker_username/icvae

Once successfully pushed, replace the command system (‘docker pull cgyan/icvae’); in ICVAE_settings.m with system (‘docker pull your_docker_username/icvae’). This allows you to easily customize your ICVAE harmonization workflow. Please note, if substantial modifications to ICVAE are required, the existing interface may not accommodate your adjustments. In such cases, you can use a Python runtime environment directly or refer to yw_Harmonization*.m. Additionally, unlike other methods, ICVAE outputs neural network parameters for each batch under the directory your/output/path/params.

#### 2.2.4 Linear

##### 2.2.4.1 Logic.

Linear regression approaches assume that the site factor has an additive impact on the data, with this influence typically following a normal distribution and being orthogonal to other potential factors. Instead of treating the site effect as a fixed effect, a linear mixed model (LMM) considers it as a random effect. This implies that the influence of the site is viewed as a variable that follows a certain probability distribution, allowing for greater flexibility and accounting for potential variability across different sites.

##### 2.2.4.2. To apply Linear.

As with other methods, the first step is to provide the file containing demographic information and SiteName. Our software offers two optional models to estimate site effects, namely general linear model and linear mixed model. To retain relevant biological variances, users can add them to the model by clicking “Adjust —->” button.

### 2.3. Output file

Output path is the directory where harmonization results will be saved. As shown inFig. 1B, the input files directly correspond to the output files, maintaining consistency in filename, type, and directory structure. This is particularly true when the “Add Dir/Sites.” For example, the input images are arranged in the format “path/to/[SiteName]/[Route]/[File.ext]” and added through “Add Sites”, the output images will be organized as “[Output dir]/[SiteName]/[Route]/[File.ext].”

An exception occurs when an organized dataset is input; in this case, the output retains the original organization, but with the prefix “Harmonized_” added to the filename to clearly distinguish it and prevent the replacement of the original file. If multiple files are imported and a consolidated file is desired, selecting the “Optional: Organize the results into one file (.csv)” option will generate an additional “HarmonizationResults.csv” file in the output directory. This file compiles all subjects’ harmonized results, with rows representing subjects and columns representing features, providing a comprehensive overview of the harmonized data for easy access in post-hoc analyses.

### 2.4. Other Features

#### 2.4.1. Input and output an organized data

The toolbox allows users to input a single file containing organized data, arranged with subjects as rows and features as columns. This file can be uploaded using the**“Add Image”**button and supports .mat, .xlsx, and .csv formats. By default, the harmonized output will be saved as a file with the same extension but prefixed with “Harmonized_” in the original filename.

Additionally, if the raw data are individual files, you can consolidate the results into a single .csv file by selecting the**“Optional: Organize the results into one file**(.csv)” option. This will generate a .csv file where the harmonized results are organized with subjects as rows and features as columns.

#### 2.4.2. Parallel workers

The**“Parallel Workers”**setting is designed for handling large datasets and complex methodologies that require extended processing times. Through parallel workers, the harmonization process can be distributed to multiple CPU cores with the MATLAB parallel computing toolbox. This option is embedded in the SMA, nonparametric ComBat/CovBat, ICVAE, and general linear model algorithms. It is not available for parametric ComBat/CovBat, as these methods are inherently fast, nor for linear mixed models, which do not support parallel processing. By default, the setting is set to 0, which means the harmonization process will run on a single core.

#### 2.4.3. Config save and load

By supporting the saving and loading of configurations, the toolbox enhances the reproducibility and replicability of scientific research.

### 2.5. Environment and installation

The harmonization module is integrated into the DPABI software and can be installed along with DPABI. It supports the Windows, macOS, and Linux operating systems. Specifically, installation can use command “addpath(genpath(your/path/to/DPABI)” or manually add subfolders of DPABI directory. To make sure it functions properly, addpath(“your/path/to/SPM”) is also needed. To those who are inaccessible to MATLAB, the stand-alone version of DPABI Harmonization is available throughhttp://rfmri.org/DPABI_Stand-Alone. You can docker it and begin harmonization.

### 2.6. Comparison to neuroHarmonize

**Fig. 6.**
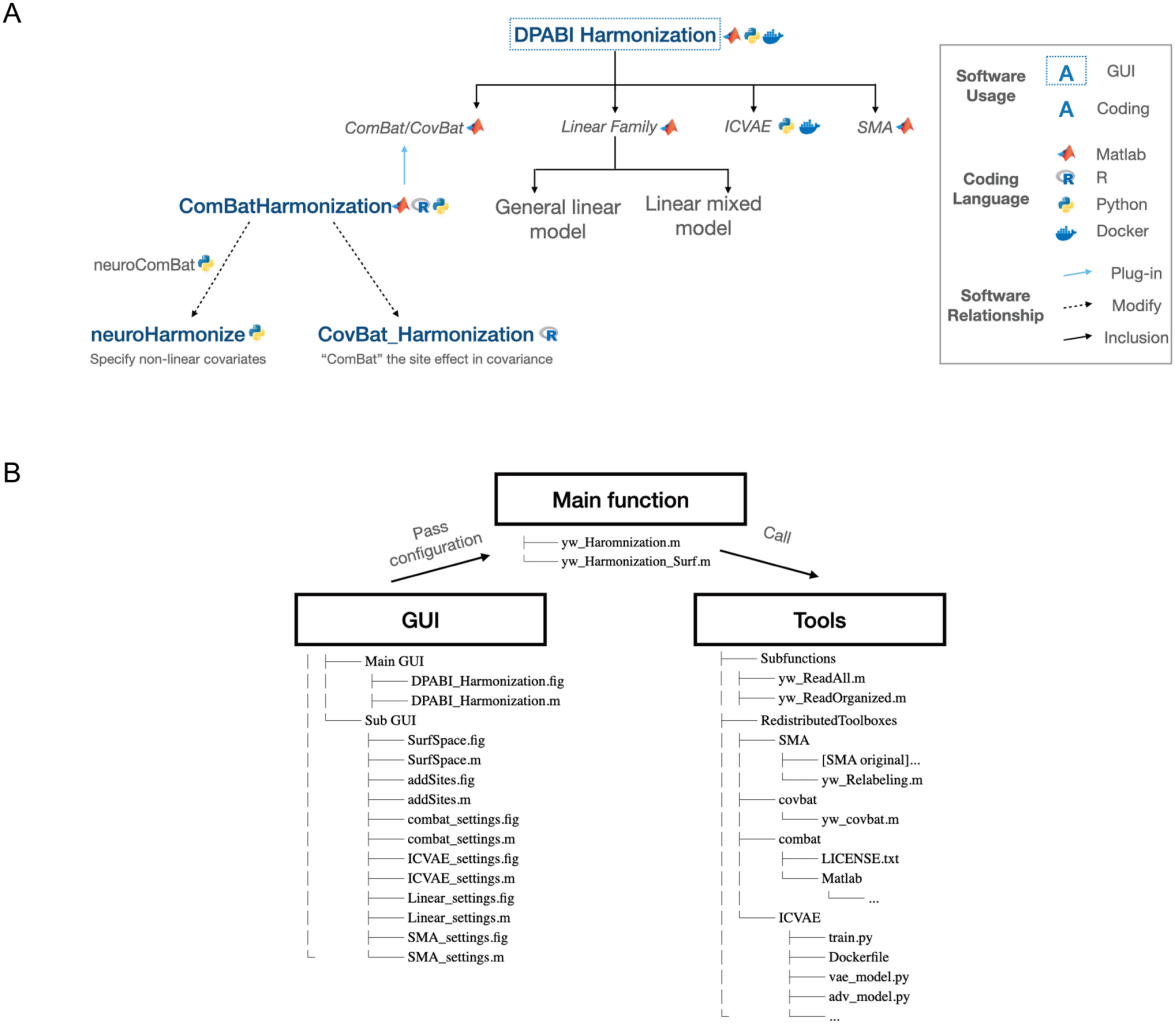
The methods of DPABI Harmonization and its relationship with other software. (A) We list ComBat-based software and compare their main features. neuroHarmonize mainly uses generalized additive models to replace the general linear model. CovBat_Harmonization harmonizes the site effect in covariance additionally with ComBat. (B) Code structure of DPABI Harmonization.

Fig. 6Aillustrates the relationships and differences between DPABI Harmonization and other softwares. Among those, neuroHarmonize (Pomponio et al., 2020) is a Python-based tool, extending the functionality of the package neuroCombat (https://github.com/Jfortin1/neuroCombat) developed by Nick Cullen (Fortin et al., 2018). It specifies covariates with generic nonlinear effects, implemented using Generalized Additive Models (GAMs) (other features seen in Github). The installation and application are based on example codes (https://github.com/rpomponio/neuroHarmonize). It provides the usage example with step-by-step elucidation of codes. However, it should be clearly noted that our new toolbox does not support nonlinear covariate effects, which is a key difference when compared with neuroHarmonize. Similar harmonization tools include ComBatHarmonization (Fortin et al., 2018;Johnson et al., 2007) and CovBatHarmonization (Chen et al., 2022). For the aforementioned non-GUI software, users are required a deeper understanding of the system and a higher level of technical knowledge and skills to operate the program effectively. Besides, they need to transfer the simplified example to their own cases, which further elevates the difficulty for application. In contrast, the simplicity of our user interface reduces the cognitive burden as well as showcases intuitive logic of different harmonization approaches. Moreover, the integration of multiple means for harmonization offers more choices.

Apart from interface utility, DPABI Harmonization incorporates the ComBatHarmonization (MATLAB version), and develops corresponding CovBat, whose results are consistent with those of R. We do not find any matches for other methods.

Fig. 6Bclearly depicts the code structure of DPABI Harmonization, which consists of three parts: users input parameters through the GUI system, the main function accepts these inputs, and various tools are called to execute the process.

## 3. Usage examples

In this tutorial, we demonstrate how to use the DPABI Harmonization tool to address site-related heterogeneity using two example datasets, each including the following steps: downloading data, installing software, inputting data and demographics, configuring methods, managing output and settings, computing time, and checking the harmonization report.

### 3.1. Step1 - Download the example data

Datasets can be downloaded fromhttp://d.rnet.co/DemoData_Harmonization.zip, andFig. 7provides an illustration of them. The CoRR dataset comprises multisite data with 420 subjects from six sites, with DPABI preprocessed ALFF results presented. The TSP-3 dataset includes 41 subjects scanned across three different scanners. Significant site effects are observed in both datasets. We use DPABI Harmonization to correct these heterogeneities for post-hoc analyses. Detailed preprocessing information can be found in the paper byWang et al. (2023).

**Fig. 7.**
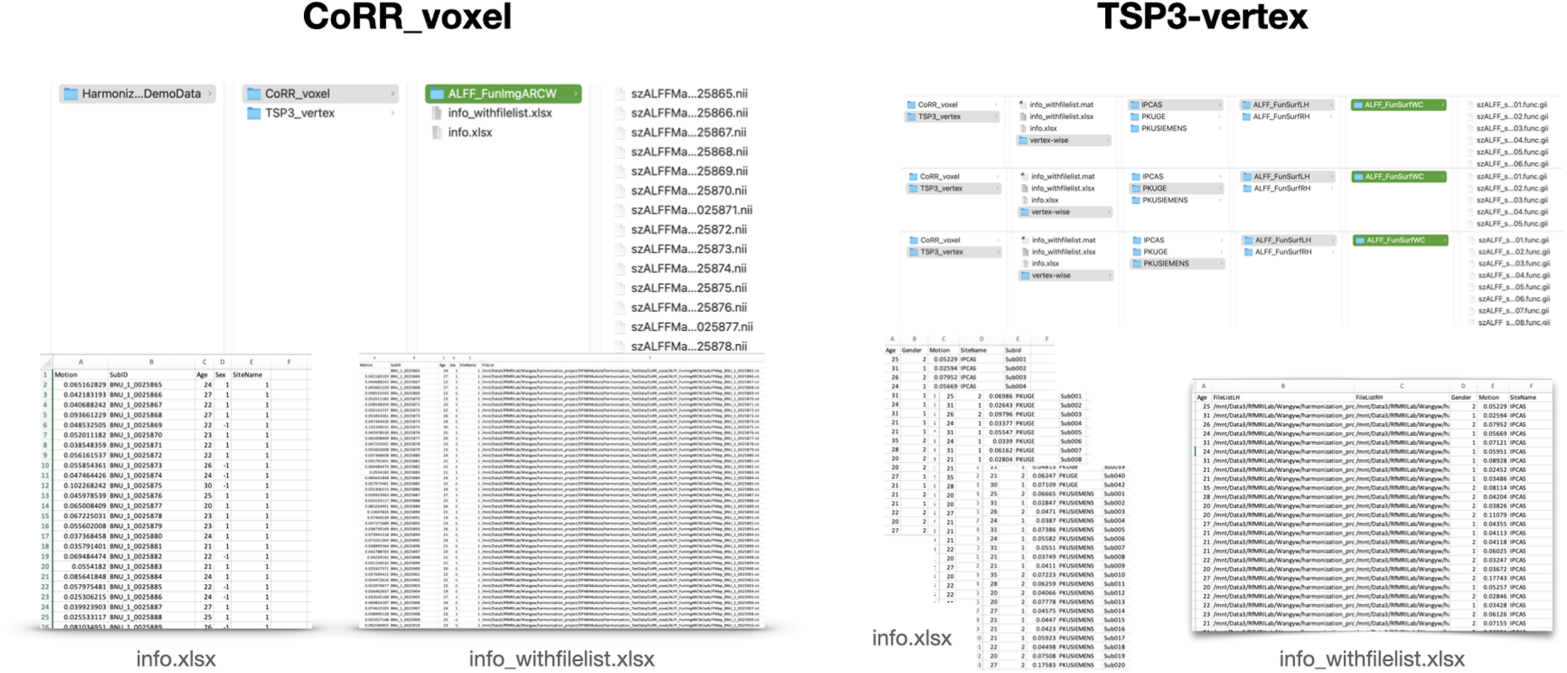
Example datasets. Left: CoRR dataset. Right: TSP-3 data. Each shows the demographics with and without the FileList column.

### 3.2. Step2 - Software download and installation

Users can find the latest version of Harmonization inhttps://github.com/Chaogan-Yan/DPABIand download it from website green “Code” button or git clone (https://github.com/Chaogan-Yan/DPABI.git). Better open your MATLAB from terminal in case you need to pull docker later for ICVAE (make sure your Docker functions well). After you unzip the downloaded package, you can input “addpath(genpath(‘your/path/to/DPABI’))” and “addpath(‘your/path/to/spm’)” in the command window of MATLAB or use “set path” interface to add DPABI to the computational environment of MATLAB.

### 3.3. Step3 - Input data and demographics

#### 3.3.1 CoRR_voxel

The dataset includes 420 .nii files organized in a single directory. There are two methods to load the data and demographics. You can follow the black arrows in the left part ofFig. 8:

**Fig. 8.**
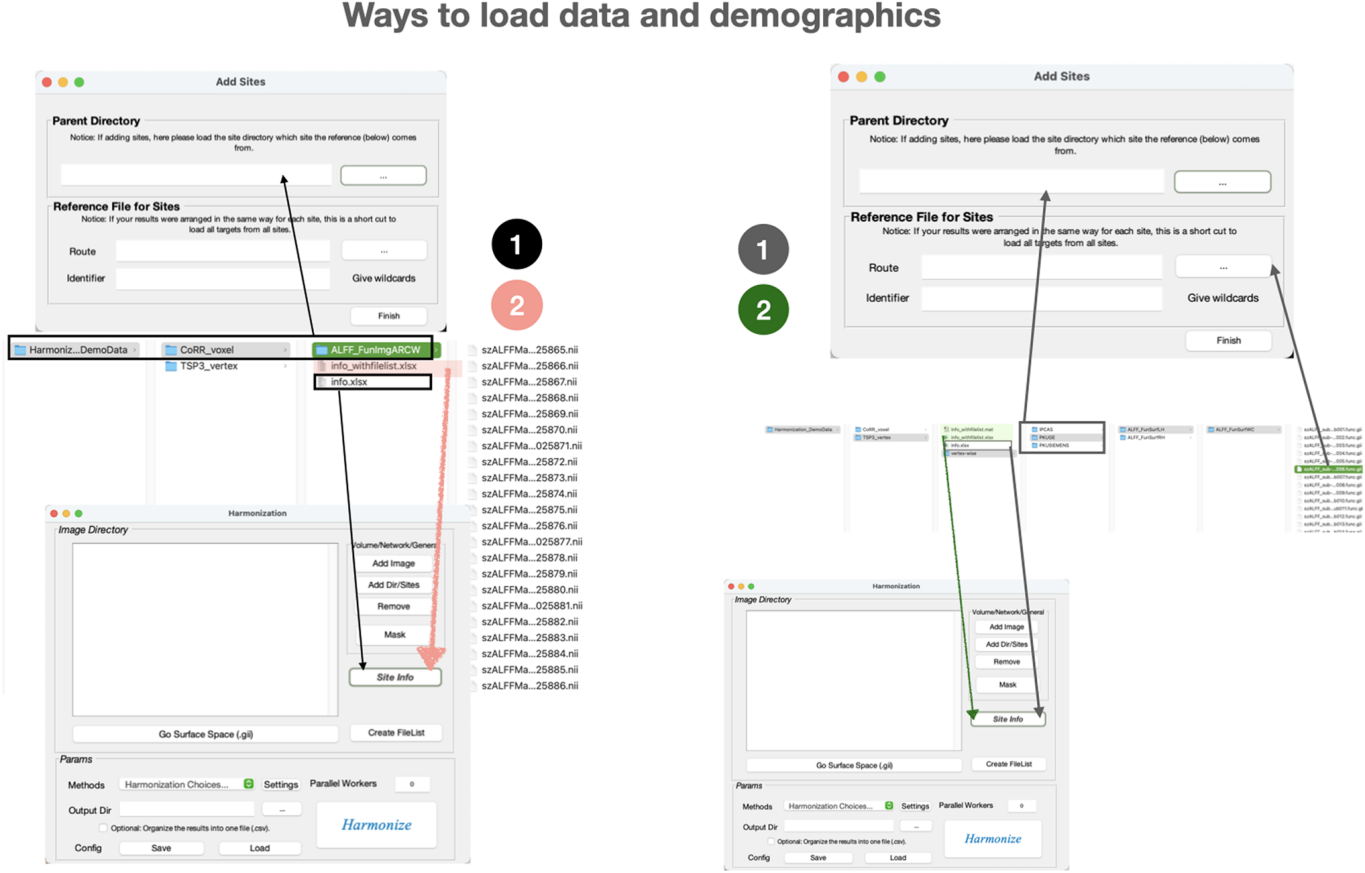
Ways to load data and demographics for exemplar datasets. Left: CoRR. Right: TSP-3. The arrows indicate where to place the content into the corresponding fields. The colors identify the different combinations of operations for loading data and demographics.

1. Use the “Add Dir/Site” option to add data to the general space. As shown in the figure, add the whole path of directory ALFF_FunImgARCW to the “Parent Directory” of Add Sites. You can either use the … button to select.
2. Click “Finish.”
3. Add the “info.xlsx” through***Site Info***button before proceeding to method settings.
4. Or just add info_withfilelist.xlsx through the***Site Info***button (refer to the pink arrow).

#### 3.3.2. TSP-3_vertex

The TSP-3 dataset consists of hemisphere surface data organized by site, with each site following the same organizational structure. To load the data, you can use the**Add Sites**button in surface space and follow the dark grey arrows in the right part ofFig. 8,

1. Choose any site as the “Parent Directory.”
2. Click the … button next to “Route” to automatically navigate to the sub-directory of the “Parent Directory”. Choose a target file for reference.
3. If the target files share the same extension, click**Finish**button. Otherwise, specify the pattern in the “Identifier” field so that the algorithm can collect them correctly.
4. Click the “**Back to Main panel”**button to add info.xlsx into***Site Info***button.

Also, you can cover all the steps above with adding info_withfilelist.xlsx/.mat through the***Site Info***button (refer to the dark green arrow).

Please note that the paths provided in the example info_withfilelist.xlsx/.mat files are placeholders and may not match your local file system. You need to update these file paths to reflect the actual full paths on your local system. Alternatively, you can use our GUI to automatically generate an info_withfilelist.xlsx by specifying an info.xlsx file. To do this, follow the instructions inFig. 8, which explain how to use the ‘Add Sites’ button to load the organized CoRR/TSP-3 data. Then, use the ‘Site Info’ button to add the info.xlsx file, and click ‘Create FileList’. This will generate a new New_info.xlsx file in the same folder as info.xlsx, which can be used as info_withfilelist.xlsx adapted for your local system. For further guidance, watch the tutorial video at:https://rfmri.org/DPABIHarmonization.

### 3.4. Step4 - Configure methods

#### 3.4.1. CoRR_voxel

We choose ComBat/CovBat method to harmonize here. When using ComBat/CovBat and Linear options, adjusting for covariates—especially the phenotypes of interest—can enhance the statistical effect. Our implementation is shown as inFig. 9(left).

**Fig. 9.**
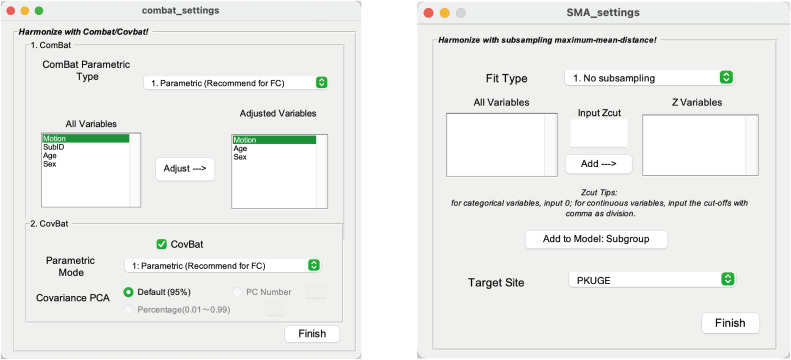
Method settings. Left: CoRR use CovBat to harmonize. Right: TSP-3 use SMA.

#### 3.4.2. TSP-3_vertex

We choose SMA method to harmonize here. Specifically, when using SMA harmonizing the data, selecting “No subsampling” is appropriate, as there are no demographic differences across the sites (Fig. 9, right).

### 3.5 Step5 - Other settings and compute time

After defining the output directory, you can choose whether to organize the results into one .csv file and save the config. Notice the output directory should be different from the input in case of cover. Configuration save can help check, reload, and share the setting. Then, click “Harmonize” and wait until the command window tells you that the harmonization and report are both finished.

Based on the size of data to be harmonized, methodology option, as well as hardware and software foundations, the runtime is uncertain. We showcase the runtime of all methods on CoRR_voxel (38810 voxels to harmonize) for reference (Table 1). The parametric ComBat/CovBat is very fast.

**Table 1:** The runtime of harmonization for various methods on example dataset.

Methods	Runtime (second)
SMA	220 (12 parallel workers)
ComBat (adjusted+nonparametric)	179 (12 parallel workers)
CovBat (adjusted+nonparametric)	180 (12 parallel workers)
ICVAE	3697 (12 parallel workers)
Linear general model	116 (12 parallel workers)
Linear mixed model	1966 (single worker)

The mixed linear model calls the table data type as its foundation; therefore, it is unable to invoke parallel cores (CPU: Intel 2.60GHz).

### 3.6 Step6 - Check the harmonization report

**Fig. 10.**
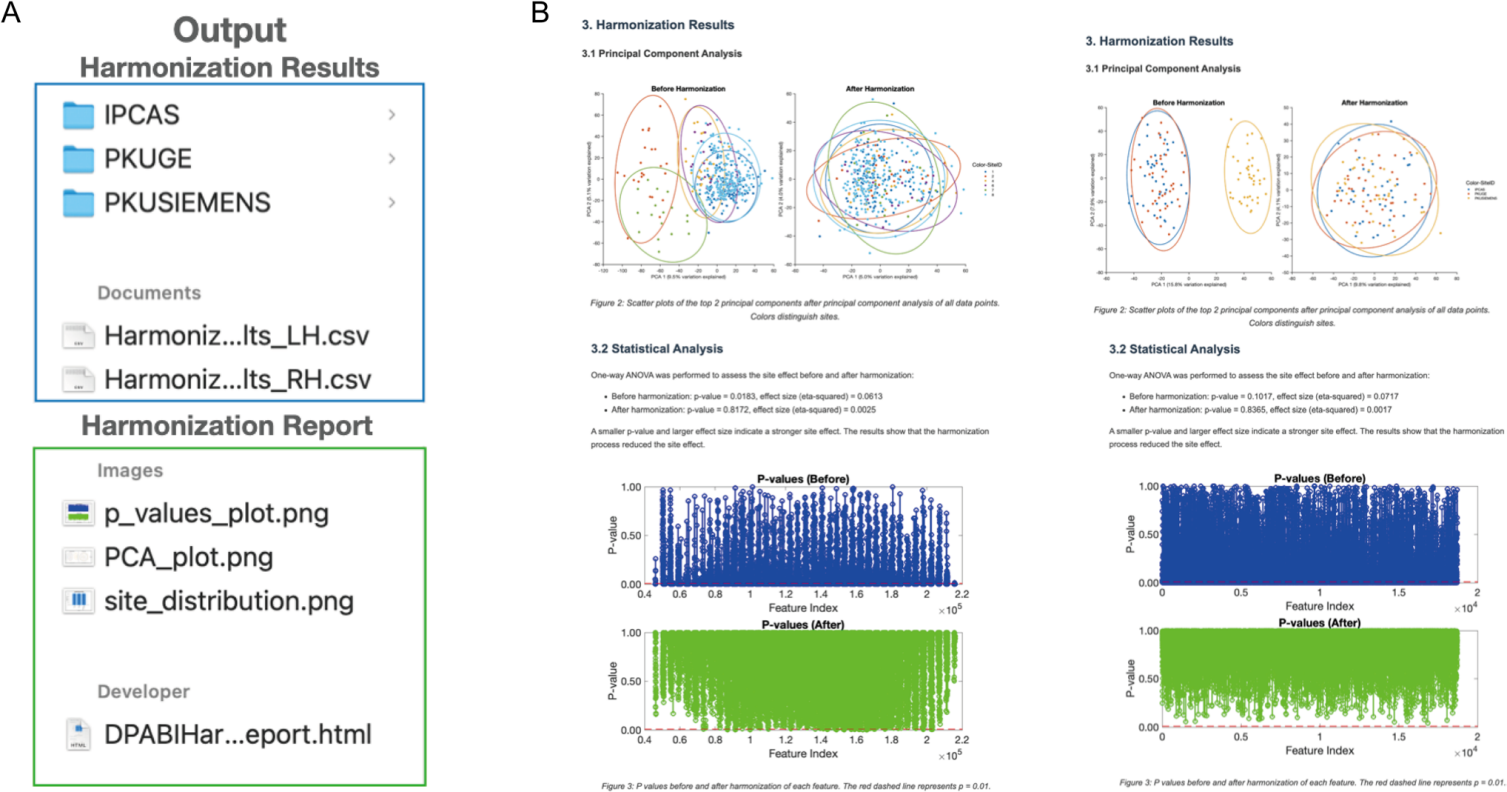
Check the harmonization reports. (A) The organization of harmonized results. (B) Left: CoRR use CovBat to harmonize. Right: TSP-3 use SMA.

Fig. 10Aillustrates the organization of harmonized results for the TSP-3 dataset. You can review the basic information of your dataset, the harmonization method used, and the preliminary examination of the transformation from unharmonized to harmonized data (Fig. 10B). Both harmonization procedures successfully remove site heterogeneity.

## 4 Discussion

### 4.1 Methodology recommendation

Based on previous studies, we recommend using SMA for harmonizing resting-state fMRI data, as it retains more biological information. CovBat can serve as a faster alternative but may preserve less biological detail. For other modalities, we encourage researchers and users to test and compare the methods to identify the most suitable approach and gain new insights.

### 4.2 Characteristics of DPABI Harmonization

1. Flexibility—To meet different input/output requirements, DPABI Harmonization supports volume-based (.nii, .nii.gz), surface-based (.gii, .gii.gz), and network-based (.mat) brain features. Additionally, users can organize their features into one .mat or .xlsx file, with subjects in row and features in column. This single file can be inputted only as its rows were ordered as a demographic file. This can make sure the harmonization process occurs correctly.
2. Coordination—DPABI Harmonization, as one module of DPABI, is empowered by volume-based preprocessing pipeline DPABI, surface-based preprocessing pipeline DPABISurf, diffusion preprocessing pipeline DPABIFiber, network analysis module DPABINet and statistical functionalities, as well as other utilities. Their coordination enables users own end-to-end analysis convenience.
3. Coherence—Based on each method’s fundamental structure, DPABI Harmonization constructs a clear interface complying with configuring logic. For example, for each harmonization method, we require users firstly to provide a demographic file which contains site labels, headed with “SiteName.” We put ComBat above CovBat under the same subGUI, in accordance with the fact that CovBat was an extension of ComBat and highly adopted its main codes. As for SMA, its main configuration consists of “Fit Type” and “Target Site.” The former decides whether there are Z variables and based on our target site selection formula, subsequently impacts the choice of the target site. This coherent design will enhance user experience and foster intuitive understanding.

### 4.3 Limitations

DPABI Harmonization is not without limitations. The offered approaches may not be the best choice for structural brain images. In the future, we shall compile new advanced techniques targeted at structural data into the Methods part. Considering application issues, while there exists an empirically-derived heuristic formula for practical use, a good standard target site is more fundamental to ensure the effectiveness of harmonization. It not only enhances the harmonization efficiency within a single study but also facilitates the seamless comparison and aggregation of various multi-site studies harmonized using SMA. To accomplish this, it is essential to establish a standardized target site with a large sample size, enriched with diverse samples from existing datasets. We are actively working toward this goal.

We offer comprehensive, step-by-step video courses free of charge online (https://rfmri.org/DPABIHarmonization) to support users in learning our software. We hope that these resources, combined with our continually updated open-source toolbox (available athttp://rfmri.org/dpabi), will serve to both novice and expert users alike. And we provide an interactive forum for users to discuss any error messages encountered:https://rfmri.org/DPABIDiscussion.

## Data Availability

The example dataset can be downloaded fromhttp://d.rnet.co/DemoData_Harmonization.zip. Users can find the latest version of Harmonization inhttps://github.com/Chaogan-Yan/DPABI. And to those who are inaccessible to MATLAB, the stand-alone version of DPABI Harmonization is available throughhttp://rfmri.org/DPABI_Stand-Alone. The relevant codes of all the harmonization methods used in the module can be found under DPABI/ Harmonization and DPABI/RedistributedTools.
